# Microalgae with Immunomodulatory Activities

**DOI:** 10.3390/md18010002

**Published:** 2019-12-18

**Authors:** Gennaro Riccio, Chiara Lauritano

**Affiliations:** Department of Marine Biotechnology, Stazione Zoologica Anton Dohrn, CAP80121 Naples, Italy

**Keywords:** microalgae, immunomodulatory activity, sulfated polysaccharides, sulfolipids, polyunsaturated fatty acid

## Abstract

Microalgae are photosynthetic microorganisms adapted to live in very different environments and showing an enormous biochemical and genetic diversity, thus representing an excellent source of new natural products with possible applications in several biotechnological sectors. Microalgae-derived compounds have shown several properties, such as anticancer, antimicrobial, anti-inflammatory, and immunomodulatory. In the last decade, compounds stimulating the immune system, both innate immune response and adaptive immune response, have been used to prevent and fight various pathologies, including cancer (cancer immunotherapy). In this review we report the microalgae that have been shown to possess immunomodulatory properties, the cells and the cellular mediators involved in the mechanisms of action and the experimental models used to test immunostimulatory activities. We also report information on fractions or pure compounds from microalgae identified as having immunostimulatory activity. Given the increasing interest in microalgae as new eco-friendly source of bioactive compounds, we also discuss their possible role as source of new classes of promising drugs to treat human pathologies.

## 1. Introduction

The potential of microalgae as source of novel drugs has recently generated a great interest for the scientific community. Microalgae are a source of several bioactive compounds such as vitamins, lipids, carbohydrates, and pigments. Several studies showed that microalgal raw extracts, fractions, and pure compounds had biological activities [1], such as anticancer [2], anti-microbial [3], anti-epilepsy [4], anti-inflammatory [5,6], and immunomodulatory activities [7]. 

The use of marine macroorganisms for drug discovery and massive production is very often limited by the sourcing and by the difficulties in culturing and scaling up [8]. On the contrary, microalgae can be easily cultivated in small or large volumes (e.g., by using photobioreactors) in order to obtain huge amount of the desired products [3,9] and their harvesting does not depend on seasons and climate. In addition, changes in cultivation conditions (i.e., light, temperature, bubbling, nutrient concentrations, salinity) may direct the synthesis of compounds of interest and increase their production [10,11,12,13,14,15].

Microalgae are adapted to live in both marine and freshwater environments, as well as in extreme conditions (e.g., hot, cold, high/low salinity, high/low light intensities, etc.,). This capability resulted in a huge diversity of species which can produce very different interesting natural products [16] with industrial and pharmaceutical interest [17]. Recent technologies have allowed to sequence genomes, metagenomes, transcriptomes, metatranscriptomes, proteomes, and metabolomes of several species or pool of species, in silico identifying gene clusters involved in the synthesis of potentially bioactive compounds and helping in the discovery of new drugs from microalgae [18,19].

Recently, various compounds from microalgae have been found to stimulate the immune system in human and murine models (e.g., modifying macrophage activations and the release of pro- and anti-inflammatory mediators [20,21,22]) and they are promising drugs with potential applications to treat human pathologies [2,7,23].

The immune system has developed a broad array of defensive mechanisms to protect the host from a high variety of pathogenic organisms and toxic substances. These mechanisms may be classified into two categories: (1) innate immune response; (2) adaptive immune response. Innate immune response acts rapidly after an invading pathogen. It involves a large number of cells and molecules, thus constitutes the first host response. Innate immune cells include basophils, dendritic cells, eosinophils, monocytes and macrophages, neutrophils and natural killer (NK) cells [24]. On the contrary, adaptive immune response involves a few number of cells and it is based on the antigen-specific receptor expressed on adaptive immune cells, T and B lymphocytes [25]. Immune system cells regulate their activities through a plethora of different mediators. Cellular mediators of these processes are cytokines, that include interleukins, tumor necrosis factor α (TNF-α), and interferon γ (INF-γ) produced predominantly by macrophages and lymphocytes [26]. In addition, another cell-signaling messenger in a wide range of physiological and pathophysiological processes [27] is nitric oxide (NO). Moreover, uncontrolled immune responses to pathogens or toxic substances can cause inflammatory tissue damages and autoimmune diseases. To prevent these effects, the magnitude of the immune response is regulated by a balance between co-stimulatory and inhibitory signals [28]. T cells are primary mediators of immune effector functions and they express multiple co-inhibitory receptors including lymphocyte-activation gene 3 (LAG-3), programmed cell death protein 1 (PD-1), and cytotoxic T-lymphocyte-associated protein 4 (CTLA-4) [28]. 

Immunomodulation includes all the processes to modify/regulate the immune response for therapeutic aims. Thus immunomodulation includes the activation of immune system to reduce inflammatory processes (e.g., immune system response to injury and infection) and fight diseases such as microbial infections and cancer (e.g., vaccination to fight infection by pathogenic agents or more recently immunotherapy to fight cancer). The last two decades have seen a crescendo of immunotherapeutic agents against tumors approved by Food and Drug Administration (FDA) [29].

The research of novel and nontoxic compounds from natural sources for cancer treatments is necessary because of toxicity and sometimes low specificity of chemotherapy and radiotherapy, as well as adverse reactions in patients. Both innate and adaptive immune system may be engaged against cancer [30,31] and there are different ways in which immune system can be used against cancer disease:

- Vaccination: traditionally, vaccination stimulated immune response using inactivated biological agents. Recent vaccines consist of highly purified synthetic macromolecules combined with adjuvant agents. Adjuvant agents potentiate the immune response by activating the antigen-presenting cells (APCs) [32,33].
- Immune checkpoint blockage: it is an innovative treatment that uses immune checkpoint inhibitors. Specific protein expressed by immune system cells or by cancer cells, such as PD-1 and PD-L1 [34,35], CTL-4 [36], STAT-3 [37] can be inhibited to induce T-cell to kill cancer cell [38].
- Monoclonal antibodies: monoclonal antibodies can be targeted against tumor specific antigens and they can trigger cell death by different mechanisms of action [39,40].
- CAR-T cells: Chimeric Antigen Receptor T-cell (CAR-T) immunotherapy consist in engineered T-cell redirected against a specific target. CAR-T therapy has shown a high rates of success (good outcome) against acute and chronic leukemia [41].

This review is focused on microalgae with immunomodulatory activity, active microalgal raw extracts, fractions, the most promising immunomodulatory compounds and the mechanisms of action, giving an overview of their pharmaceutical potential.

## 2. Microalgae with Anti-Inflammatory Activity 

Anti-inflammatory properties were previously found for various marine diatoms, such as Porosira glacialis, *Attheya longicornis* [42], *Cylindrotheca closterium*, *Odontella mobiliensis*, *Pseudonitzschia pseudodelicatissima* [43] and *Phaeodactylum tricornutum* [44], for the dinoflagellate *Amphidinium carterae* [44] and the green algae *Dunaliella bardawil* and *Dunaliella tertiolecta* [45]. Lauritano et al. 2016 [43] and Ingebrigstein et al. [42] evaluated the release of one of the main effectors of inflammation, the tumor necrosis factor α (TNFα) [46] in lipopolysaccharide (LPS)-stimulated monocytic leukemia cells (THP-1). Samarakoon et al. [44] tested the inhibition of nitric oxide (NO) production (%) level, one of the inflammatory mediators, as positive evidence of anti-inflammatory activity on LPS-induced RAW macrophages. 

In a double-blind placebo-controlled randomized clinical trials, seventy patients, affected by non-alcoholic fatty liver disease (NAFLD) were recruited to test the effects of *Chlorella vulgaris*. 300 mg tablet commercially available as ALGOMED® of *C. vulgaris* supplements contained 98% *C. vulgaris* powder, 1% separating agent, 1% plant-based magnesium stearate were administered. For eight weeks, patients received four tablets/day of *C. vulgaris*. The patients treated with *C. vulgaris* tablets had significantly lower serum levels of the pro-inflammatory cytokine TNF-α [47].

Finally, Lavy et al. 2003 [45], showed protective effects of spray-dried *D. bardawil* powder against acetic acid-induced small bowel inflammation in rats, while Caroprese et al. [48] showed that a mixture of phytosterols from *D. tertiolecta* reduced the cytokine production in a sheep model of inflammation. Except for the last case, no much information is available on the compounds responsible for the anti-inflammatory activity observed in the mentioned studies.

In addition, there are some studies reporting the anti-inflammatory activity of pure compounds isolated from both marine and freshwater microalgae: the carotenoids lutein and astaxanthin, the fatty acids EPA (eicosapentaenoic acid) and DHA (docosahexaenoic acid), and sulphated polysaccharides (sPS) [5].

Ingebrigtsen et al. and Lauritano et al. also used an OSMAC (one strain-many compounds) approach changing culturing condition parameters, such as nutrients [43], light, and temperature [42], in order to trigger anti-inflammatory activity. They observed that the studied species were active only in specific conditions highlighting the importance of selecting the proper parameters to increase bioactivity of microalgal extracts. Similarly, Montero-Lobato et al. (2018) [5] reported the chemical triggers (e.g., low oxygen, NaCl and nutrient starvation) to increase the production of anti-inflammatory molecules (i.e., carotenoids, fatty acids, and sulphated polysaccharides) from some microalgal species (e.g., *Chlorella zofingiensis, Coccomyxa onubensis*, *Cromochloris zofingiensis*, *Dunaliella salina*, *D. tertiolecta*, *Haematococcus pluvialis*, *Nannochloropsis oceanica*, *Pavlova lutheri*, *P. tricornutum*, and *Spirulina platensis*). 

## 3. Microalgae with Immunomodulatory Activity

To date several microalgae have been found to have immunomodulatory activity in human or in other organisms (i.e., sheep and mouse); however, the compounds responsible for this activity are often still unknown. As reported in Table 1, dried algae and raw extracts were active against several immune cells. Cutignano et al. [49] tested raw methanolic extracts and fractions derived from them (named A-E) of various microalgal species (i.e., *Alexandrium andersoni FE108*, *Alexandrium tamarense FE107*, *Chaetoceros calcitrans FE20*, *C. socialis FE17*, *Ditylum brightwellii*, *D. salina FE209*, *Skeletonema costatum RCC1716*, *S. dohrnii FE82*, *S. marinoi FE65*, *S. pseudo-costatum FE25*, *Skeletonema* sp. KS5 and *Thalassiosira weissflogii 1336*) on human peripheral blood mononuclear cells (PBMCs). Immunostimulant activity in this study was considered as induction of IL-6 in PBMCs cells. They found that raw methanolic extracts were active for *S. costatum* and *S. dohrni.* Fractionation allowed for these two species to identify the active fractions. Triglycerides rich fraction (named fraction E) was active for *S. costatum*, while nucleoside rich fraction (named fraction B) was active for *S. dohrnii.* For other species whose raw methanolic extracts were not active, fractionation also helped to identify active fractions. This is probably due to the fact that fractions contained less salt (mainly NaCl) than primary extracts. In particular, glycololipidic and phospholipidic fraction (named fraction C) was active for *A. tamarense*, nucleosides rich fraction (named fraction B) was active for *D. salina,* amminoacids and saccarides rich fraction (named fraction A) was active for *C. calcitrans*, while glycolipidic and phospholipidic fraction (fraction C) and the fraction rich in free fatty acids and sterols (named fraction D) were active for *T. weissflogii.*

Other extracts derived from microalgae have been tested to evaluate their immunostimulatory activity. *Chlorella stigmatophora* polysaccharide extracts (aqueous extracts) have been tested in in vitro and in vivo mouse models. In particular, the BALB/c mice were injected with 5 mg of polysaccharide extracts per kg of body weight in order to test the phagocytic activity by using macrophages from peritoneal cavity both for in vitro and in vivo assays. The C57BI mice were injected with polysaccharide extracts at 5 mg or 10 mg per kg of body weight, two days before or two days after the injection of sheep red blood cells (SRBC). This experiment was performed in order to test the activation on SRBC [50]. Both experiments showed that *C. stigmatophora* polysaccharide extracts were able to activate phagocytic activity of macrophages from the peritoneal cavity.

*Euglena gracilis* β-glucans (called paramylon) at 150 µg/ml activated NK cells and increased the levels of the two pro-inflammatory mediators TNF-α and IL-6 [51,52]. *Gyrodinium impudicum* KG03 sulfated exopolysaccharides (at 0.1–10 µg/ml) induced cellular response in peritoneal macrophages in in vitro murine models [53]. In addition, sulphated polysaccharides extracted from *Tribonema* sp have been found to enhance macrophage cell viability and improve the expression of cytokines. Authors found that cell viability was only improved in the presence of 25 µg/mL of sulfated polysaccharides, while the cytokine expression increased with sulfated polysaccharide treatment at 12.5–200 µg/mL [2]. Park et al. [54] tested the immunostimulatory activity of *Thraustochytriidae* sp. on human lymphocyte B-cells. They found that the polysaccharides from this alga tested at 10^−3^ to 10^−9^ w/v were able to stimulate cell proliferation but not cytokine production.

Double-blind placebo-controlled randomized clinical trials have also been performed to test the in vivo immunostimulatory activity of *C. vulgaris*. Sixty people were recruited and randomly assigned to placebo group or *Chlorella* group. Tested pills contained dried *C. vulgaris* as active ingredient (Daesang Corp., Seoul, Korea). The diet was supplemented with 5 g/day of *C. vulgaris*. All participants were encouraged to maintain their usual lifestyle and dietary habits. *C. vulgaris*-supplemented diet improved NK activity from PBMCs isolated from the treated patients and increased the serum level of INF-γ, IL-1β and IL-12 [55].

Microalgae food supplementation has also been associated to immunostimulatory activity. Indeed, diet supplementation of commercially available spray-dried preparations of *D. salina* (369 or 922.5 mg of algal extract per kg of body weight) in mice improved NK and macrophage activation, as well as survival rate of leukemic mice [56]. Orally administration of *Tetraselmis chuii* (50 or 100 g of lyophilized alga per kg of dried food) in gilthead seabream (*Sparus aurata* L) induced an increase in expression levels of several genes associated to immune system, such as T-cell receptor beta (TCR-β), major histocompatibility complex genes and IgM [57]. 

Most of these compounds act as vaccine adjuvants improving the immune response by activating APCs [7,49,58]. In Table 1, we have summarized the algae which have shown immunostimulatory activity. Pure compounds from microalgae with immunomodulatory activity will be discussed in the next paragraph.

## 4. Immunomodulatory Compounds from Microalgae

### 4.1. Sulfate Polysaccharides

Microalgae are known to be excellent producers of polysaccharides (PS), including exocellular polysaccharides (EPS) and sulphated polysaccharides (sPS). PS have been shown to be good antiviral agents, health foods, antioxidants, anti-inflammatory, and immunomodulatory compounds, and they can also be used as lubricants for bone joints and drag-reducing substances for ships [62]. sPS from marine sources exhibited, in particular, immunomodulatory activities [2,63,64,65,66,67,68,69], and are promising candidates for drug development. sSP have been found in several macroalgae and microalgae, in both marine and freshwater environments, such as in the green alga *Monostroma nitidum* [70], the red algae *Gelidum corneum* [67] and *Gracilaria caudata* [71], the diatom *P. tricornutum* [23], the dinoflagellate *G. impudicum,* the freshwater xanthophycea *Tribonema* sp. [2] and others [58].

Regarding the mechanisms of action evaluated for sPS extracted from microalgae few studies have reported details. In particular, Chen et al. [2] and Bahramzadah et al. [68] tested the immunostimulatory effects of sPS extracted from *Tribonema* sp. (12.5–200 μg/mL or 10–50 μg/mL respectively) on RAW 264.7 murine macrophage cells. The results of these two independent studies showed a macrophage cell stimulation characterized by the up-regulation of interleukin-6 (IL-6), interleukin-10 (IL10), and tumor necrosis factor alpha (TNF-α) levels after 24 h of sPS treatment. Bae et al. [53] tested the activity of the sPS from the dinoflagellate *G. impudicum* on murine peritoneal macrophages. In particular, murine macrophages were co-incubated with B16 mouse melanoma cells in the absence and in the presence of sPS. sPS increased cytotoxicity in macrophages and there was an increase in nitric oxide production but not in IL-1, IL-6, and TNF-α expression.

### 4.2. Sulfolipids

**Figure 1:**
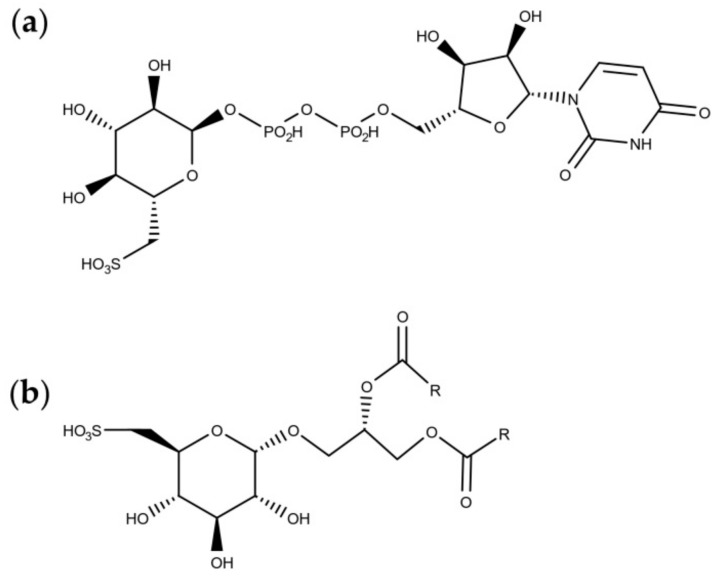
Molecular structures of (**a**) sulfoquinovose, and (**b**) sulfoquinovosyldiacylglycerols.

Sulfolipids are constituents of the thylakoid in plant and algal chloroplasts [72] and they constitute the anionic fraction of the mono- and digalactosyl-diacylglycerols [73]. Sulfoquinovose (Figure 1a), the building block of sulfolipids (Figure 1b), is also the major component of the biological sulphur cycle [74] and it is produced by photosynthetic organisms at a rate of 1010 tons per year [75]. 

Sulfolipids have been found to have several potential activities to treat and prevent human pathologies. For instance, it was demonstrated that they are potent glutaminyl cyclase inhibitors, with possible applications against Alzheimer’s disease [76]. In addition, sulfolipids have been shown to be able to activate the immune system, with application as vaccine adjuvants [7]. In particular, Manzo et al. [7] used microalgae-derived sulfolipids as lead compounds to generate a synthetic sulfoglycolipid (called β-SQDG18) as vaccine adjuvant to trigger a more effective dendritic cell (DC) activation and improve immune response against cancer cell (i.e., B16 mouse melanoma cells). β-SQDG18, tested at 0.01 to 10 μg/mL on DCs, increased the percentage of CD83-positive DCs, stimulated the production of the pro-inflammatory cytokines IL-12 and INF-γ and increased the expression levels of IL-1α, IL-1β, IL-18, and IL-27 after 24 h exposure. β-SQDG18 was also tested at 25 mg via subcutaneous injection in a model of cancer vaccine against B16 mouse melanoma cells, the synthetic sulfolipids were able to reduce tumor growth and induce expansion of both lymphocytes and APCs memory in the treated mice, thus resulting a good adjuvant in cancer vaccination. This synthetic sulfolipids was patented as Sulfavant (EP3007725A1, WO2014199297A1). Moreover, the inventors improved the sulfavant synthesis and modified it producing two epimers named Salfavant-S and Sulfavant-R, that induced a more effective immune response (increase in IL-12 and INF-γ levels) [77].

### 4.3. Polyunsaturated Fatty Acids (PUFAs)

**Figure 2:**
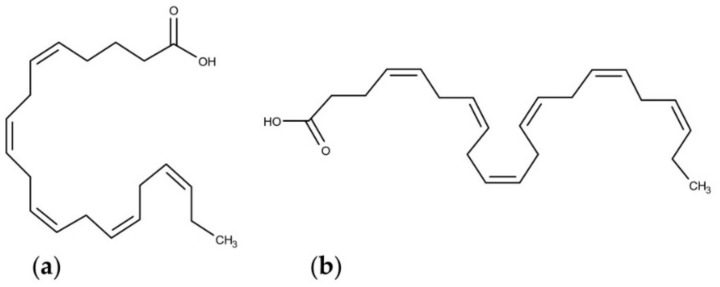
Molecular structures of (**a**) eicosapentaeonic acid (EPA) and (**b**) docosahexaeonic acid (DHA).

PUFAs are fatty acids that contain two or more double bonds in their carbon chain. There are two well-known classes of PUFA, namely omega-6 (ω-6) and omega-3 (ω-3) series. The position of the first unsaturation on carbon backbone from the methyl, omega-C, generated the name of the two different classes. Microalgae are important source of polyunsaturated fatty acids and they are able to synthesize both omega 6 and omega 3 fatty acids and several species of microalgae have been characterized for their PUFA production (Table 2). Considering the continuous reduction of available fishery and seafood resources and the increasing market request of vegan products, microalgae have been considered as valuable alternatives, as well as sustainable and eco-friendly PUFA producers [78]. The omega 6 fatty acids class include γ-linoleic acid (GLA) and arachidonic acid (ARA), the omega 3 fatty acid class include eicosapentaeonic acid (EPA), known as the most abundant PUFA in phytoplankton [79], and docosahexaeonic acid (DHA) (Figure 2) [80]. Diatoms are the principal omega 3 fatty acid producing algae, especially EPA and DHA [81]. In Table 2, we report the microalgae for which the most abundant PUFAs produced were studied.

Several biological functions have been associated to DHA and EPA, such as a wide range of beneficial cardiovascular effects [82], antimicrobial activities [83], anti-inflammatory modulation [84], antioxidant properties [85], beneficial effects on respiratory diseases [86,87], antitumoral activity [88], and inflammation attenuation in adipose tissues [89]. Different authors highlighted the immune-stimulatory properties of PUFA [90,91], as well as their anti-inflammatory properties [92].

Han et al. [93] tested the effects of EPA on macrophage RAW264.7 cell cultures. EPA increased macrophage proliferation rate, induced the release of nitric oxide and cytokines such as IL-1β, IL-6, TNF-α, and INF-γ through the activation of GPR120-mediated Raf kinases mitogen-activated protein kinases p44/p42 (ERK1/2)-inhibitor of nuclear factor kappa-B kinase subunit β (IKKβ)-nuclear factor kappa B p65 (NF-kB) signaling pathways. In this study, macrophage RAW264.7 cells were incubated in the presence of 0.6 to 3 μM EPA and their activation was analyzed after 24 h. DHA were found to modulate dendritic cell activities through improved expression of peroxisome proliferator-activated receptor gamma (PPAR γ). After 24 h, DHA altered the mature DC phenotype, increased macrophage inflammatory proteins 1α (MIP-1α) chemotaxis in immature DCs, and reduced the levels of IL-6, IL-10, and IL-12 in DCs [91]. After 6 days, DHA modulated the differentiation of immature DCs from monocytes (rise in the DC markers CD36, CD83, and CD86). 

### 4.4. Astaxanthin

Another important microalga-derived compound is astaxanthin (ASX). ASX, a carotenoid pigment, has been found in several freshwater and marine microorganisms, including bacteria, yeast, fungi, and microalgae, as well as in some aquatic organisms that feed on them. ASX is known to have antioxidant [115], anti-inflammatory [116], anti-obesity [117], cardiovascular [118] and anti-proliferative activities [1]. The use of ASX-containing products as human health food supplements is increased in the last years. In 2018, ASX had a market size of over US$100 million [119]. ASX became an important compound in the global market [120] and its safety and uses have been widely discussed [119]. The commercially available astaxanthin approved by the Food and Drug Administration (FDA) is mainly derived from the microalga *Haematococcus pluvialis*, even if other cases are reported as well (e.g. from the bacterium *Paracoccus carotinifaciens*) [119]. It has a plethora of applications, such as eye health and vision [119], skin health [121] and body exercise performance and recovery [119]. In addition, it has applications in the treatment of Parkinson disease [122] and cancer [1].

ASX has been also found to enhance immune response in several studies. Park et al. [123] tested the effect of ASX in a randomized double-blind, controlled study. ASX was administered for 8 weeks at 2 or 8 mg/d. ASX increased natural killer cell cytotoxic activity, and increased the total T and B cell subpopulations. ASX has been found to increase the lymphocyte proliferation in in vitro mice model and increase the levels of IL-2 and INF-γ in ex vivo mice model [124]. Moreover, Davinelli et al. [125] tested the activity of ASX in mice model after *Helicobacter pylori* inoculation. The splenocytes from treated mice were isolated and the production of the cytokines involved in immune response to *H. pylori* was evaluated. The levels of the IL-2, IL-10, and INF-γ increased after 6 weeks of treatment.

## 5. Discussion 

Natural products from marine and freshwater microalgae have been widely studied in the last years highlighting their diversified activities, such as antioxidant, antibacterial, antitumor, anti-inflammatory, anti-hypertensive, cardioprotective, and antidiabetic [126,127]. In addition, various studies have used an OSMAC approach in order to increase the production of specific metabolites [5,42,43]. Several active raw extracts, fractions, and pure compounds have been identified, however, they have not reached the clinical trials yet (https://www.midwestern.edu/departments/marinepharmacology/clinical-pipeline.xml). On the contrary, microalgal extracts and compounds have already found application in the nutraceutical and cosmetical sectors [128,129,130,131,132,133,134,135]. 

Microalgae are, in fact, a rich resource of macronutrients and therefore, they have been proposed as a potential sustainable food source [128,129,130]. Recently, Neumann et al. [128] also analyzed nutrient contents of the microalgae *Chlorella vulgaris*, *Nannochloropsis oceanica, P. tricornutum* and tested their food safety in mice. The microalgal species most used as food supplements are *Chlorella* sp., *D. tertiolecta*, and *D. salina* [135]. In addition, *D. tertiolecta* and *Tetraselmis suecica* have been widely used as source of vitamin E for cosmetic formulation, and *D. salina* has been also used for the high content β-cryptoxanthin, which induces hyaluronic acid synthesis [134]. 

The immunomodulatory activity of microalgae has been reported only recently, and several active extracts or fractions have been identified as well as pure compounds. Further analyses have been performed to identify the optimal active concentrations, their mechanisms of action and possible applications.

In the last decades, immunotherapy has proved to be an efficient weapon against cancer, indeed several immunotherapies have been developed and placed in the market [136,137]. Recent findings have allowed to actively use the immune system cells [28,138]. In addition DCs have been investigated for the preparation of DC-based vaccine against tumor [139,140]. DCs are the most efficient APCs [141,142] and are also called “nature’s adjuvant” to induce activation and specific expansion of cytotoxic T lymphocytes [143]. However, the costs of these innovative treatments are high, becoming a limiting factor for immunotherapy [144].

**Figure 3:**
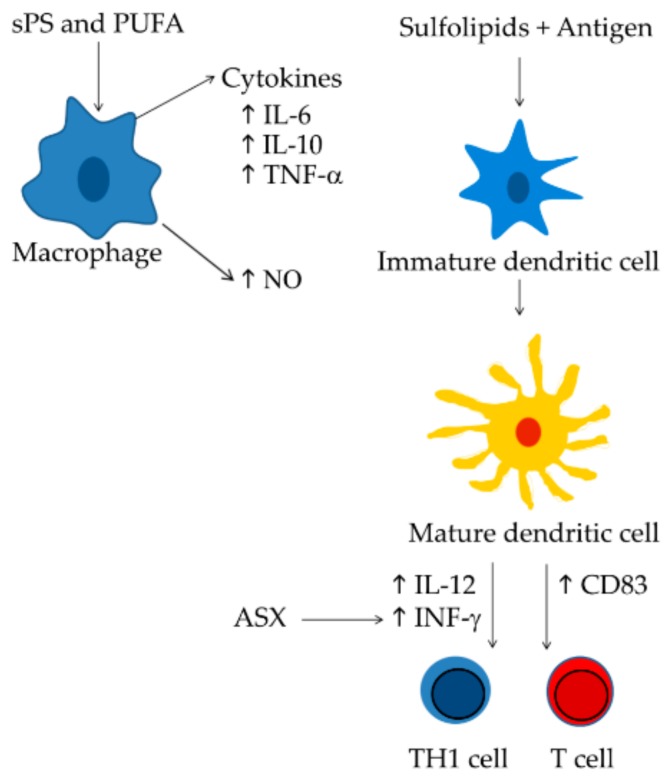
Mechanisms of action of the most promising immunostimulatory compounds. Sulphate polysaccharides and polyunsaturated fatty acids trigger macrophage activation increasing cytokine and NO levels [2,53,93]; Sulfolipids increase the number of CD83-positive cells associate to mature dendritic cells [7], in turn dendritic cells generate active T cells [143] and prime an efficient Th1 cell response [154]. Astaxanthin improves T-cell response increasing INF-γ levels [123].

A good alternative to reduce the costs of the current treatments could be the production of immune-stimulatory compounds from marine sources and microalgae appeared to be excellent candidates. Different classes of microalgal-derived compounds have shown immune-stimulatory activity, such as sulfo-polysaccharides, sulfated lipids, polyunsaturated fatty acids, and astaxanthin. All these compounds are able to stimulate macrophage cells [2,53], T-cell [56,123], or dendritic cells [7,77,91] (Figure 3). In addition, they are the most promising compounds that act as molecular adjuvants [58,77] and are able to stimulate DC maturation and specific immune responses. 

Nevertheless, the interesting properties of microalgae for human health applications have increased the exploration and exploitation of a plethora of possible source environments. Recent European Union-funded projects, under both the Research and Innovation funding programmes 7th Framework Programme and Horizon 2020 (FP7 and H2020 programs, respectively), focused on microalgal bioactivities and their possible market applications (e.g., EMBRIC, GIAVAP, PharmaSea and SUNBIOPATH) [145]. 

**Figure 4:**
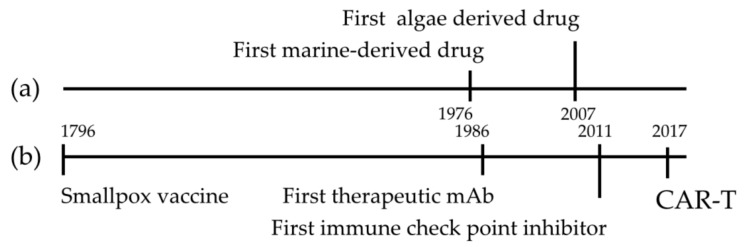
Development of immunotherapy and marine derived drugs. The timeline highlights Food and Drug Administration (FDA) approval in the field of marine derived drugs discovery (**a**) and immunotherapy (**b**).

Marine-derived compounds discovery is a young branch of the science in comparison with immunotherapy (Figure 4). The first vaccine was developed in 1769 [146] and about two thousand years later the first marine derived drug was approved [147]. But the rapid development and approval of new immune-therapeutic strategies [148,149] and the large amount of new compounds from microalgae [1,147,150], may lead to the finding of new promising drugs for immunotherapy. The number of microalgal species producing immunomodulatory molecules has not yet been adequately characterized, and considering their huge chemical diversity, eco-friendly approach for sampling and easy culturing compared to macroorganisms, they could be promising new sources of immunomodulatory molecules [151]. Finally, the application of new bioengineering tools, such as CRISP/Cas9 system for gene editing and gene knock-out in marine algae, [152,153] could be very promising in order to improve the production of microalgae enriched in molecules with immunomodulatory activity.

## Figures and Tables

**Table 1:** Marine microalgae synthesizing immunomodulatory compounds or possessing immunomodulatory properties. IL is for interleukin, PBMC is for human peripheral blood mononuclear cells, NA is for not available, w/v is for weight/volume.

Microalgae	Extract/Fraction/Compound	Active Concentration	Mechanism/ Organism and Target Cells (or Model)	Reference
*Alexandrium tamarense*	Total Extract/Fractions	NA	Activation of IL-6/Human PBMC	[49]
*Chaetoceros calcitrans*	Fractions	NA	Activation of IL-6/Human PBMC	[49]
*Chaetoceros socialis*	Total extract	NA	Activation of IL-6/Human PBMC	[49]
*Chlorella stigmatophora*	Crude polysaccharide extracts	5 or 10 mg/kg	Activation of phagocytic activity - SRBC/Mouse	[50]
*Chlorella vulgaris*	Diet supplementation/ commercially available pills	5 g/day	Improve of NK activity and serum level of INF-γ, IL-1β and IL-12/Human trials	[55]
*Dunaliella salina*	Diet supplementation of commercially available spray-dried preparations	369, and 922.5 mg/kg	MiceNK and Macrophage activation/In vivo mice model	[56] [49]
*Dunaliella salina*	Fractions	NA	Activation of IL-6/Human PBMC	[49]
*Euglena gracilis*	β-Glucans	150 μg/mL	Activation of NK cells and improve in inflammatory mediator/Human PBMC	[51,52]
*Gyrodinium impudicum*	Sulfated exopolysaccharides	0.1–10 μg/mL	Macrophage activation/Murine	[53,59]
*Skeletonema costatum*	Total Extract/Fractions	NA	Activation of IL-6/Human PBMC	[49]
*Skeletonema dohrnii*	Total Extract/Fractions	NA	Activation of IL-6/Human PBMC	[49]
*Skeletonema marinoi*	Total Extract	NA	Activation of IL-6/Human PBMC	[49]
*Spirulina* sp.	Food supplement of condensed water-soluble extract of commercially available spray dried *Spirulina*	NA	Augmentation of interferon production and NK cytotoxicity /Human trials	[60,61]
*Tetraselmis chuii*	Orally administration of lyophilized microalgae	50 or 100 g/Kg	Increase in hemolytic complement activity, phagocytic capacity and expression levels of β-defensin, major histocompatibility complex IIα and colony-stimulating factor receptor-1/Gilthead sea bream	[57]
*Thalassiosira weissflogii*	Fractions		Activation of IL-6/Human PBMC	[49]
*Thraustochytriidae* sp.	Exopolysaccharides	10^−3^ to 10^−9^ w/v	B-cells proliferation/Human	[54]
*Tribonema* sp.	Sulfated polysaccharides	12.5–200 μg/mL	Macrophage proliferation and improved expression of cytokines/Mouse	[2]

**Table 2:** Main polyunsaturated fatty acids (PUFAs) identified in selected microalgae. The table shows only the microalgae for which the most abundant PUFAs produced were reported, and corresponding references.

Microalgae	PUFA	Ref
*Amphidinium carterae*	ARA/EPA	[94]
*Amphiprora hyaline*	EPA	[95]
*Amphora coffeaeformis*	EPA/DHA	[96]
*Chlamydomonas reinhardtii*	ω-3	[97]
*Chlorella minutissima*	EPA	[98]
*Cocconeis scutellum*	ω-3/ω-6	[99]
*Conticribra weissflogii*	EPA/DHA	[96]
*Cryptomonas* sp.	EPA/DHA	[100]
*Cylindrotheca fusiformis*	EPA	[95]
*Fragilaria pinnata*	EPA	[95]
*Isochrysis galbana*	EPA/DHA	[101]
*Karlodinium veneficum*	ω-3	[102]
*Nannochloropsis gaditana*	EPA	[103]
*Nannochloropsis* sp.	EPA	[104]
*Nitzschia closterium*	EPA	[95]
*Nitzschia laevis*	ARA/EPA	[105]
*Nitzschia plea*	EPA	[106]
*Parietochloris incisa*	ARA/EPA	[94]
*Pavlova lutheri*	EPA/DHA	[107]
*Phaeodactylum tricornutum*	EPA	[108]
*Pleurochrysis carterae*	ω-3	[109]
*Porphyridium purpureum*	ARA/EPA	[110]
*Proschkinia complanatoides*	EPA	[111]
*Rhizosolenia setigera*	EPA	[95]
*Rhodomonas* sp.	EPA/DHA	[100]
*Skeletonema costatum*	EPA	[112]
*Skeletonema marinoi*	EPA	[113]
*Storeatula major*	ω-3	[102]
*Tetraselmis suecica*	EPA	[114]
*Thalassionema nitzschioides*	EPA	[95]
*Thalassiosira pseudonana*	ARA/EPA	[94]
*Thalassiosira stellaris*	EPA	[95]
*Thalassiothrix heteromorpha*	EPA	[95]

## References

[1] Martinez Andrade K.A., Lauritano C., Romano G., Ianora A. (2018). Marine Microalgae with Anti-Cancer Properties. Mar. Drugs.

[2] Chen X., Song L., Wang H., Liu S., Yu H., Wang X., Li R., Liu T., Li P. (2019). Partial Characterization, the Immune Modulation and Anticancer Activities of Sulfated Polysaccharides from Filamentous Microalgae *Tribonema* sp.. Molecules.

[3] Martinez K.A., Lauritano C., Druka D., Romano G., Grohmann T., Jaspars M., Martin J., Diaz C., Cautain B., de la Cruz M. (2019). Amphidinol 22, a New Cytotoxic and Antifungal Amphidinol from the Dinoflagellate *Amphidinium carterae*. Mar. Drugs.

[4] Brillatz T., Lauritano C., Jacmin M., Khamma S., Marcourt L., Righi D., Romano G., Esposito F., Ianora A., Queiroz E.F. (2018). Zebrafish-based identification of the antiseizure nucleoside inosine from the marine diatom *Skeletonema marinoi*. PLoS ONE.

[5] Montero-Lobato Z., Vazquez M., Navarro F., Fuentes J.L., Bermejo E., Garbayo I., Vilchez C., Cuaresma M. (2018). Chemically-Induced Production of Anti-Inflammatory Molecules in Microalgae. Mar. Drugs.

[6] Rodriguez-Luna A., Avila-Roman J., Gonzalez-Rodriguez M.L., Cozar M.J., Rabasco A.M., Motilva V., Talero E. (2018). Fucoxanthin-Containing Cream Prevents Epidermal Hyperplasia and UVB-Induced Skin Erythema in Mice. Mar. Drugs.

[7] Manzo E., Cutignano A., Pagano D., Gallo C., Barra G., Nuzzo G., Sansone C., Ianora A., Urbanek K., Fenoglio D. (2017). A new marine-derived sulfoglycolipid triggers dendritic cell activation and immune adjuvant response. Sci. Rep..

[8] Malve H. (2016). Exploring the ocean for new drug developments: Marine pharmacology. J. Pharm. Bioallied Sci..

[9] Ugwu C.U., Aoyagi H., Uchiyama H. (2008). Photobioreactors for mass cultivation of algae. Bioresour. Technol..

[10] Chrismadha T., Borowitzka M.A. (1994). Effect of Cell-Density and Irradiance on Growth, Proximate Composition and Eicosapentaenoic Acid Production of *Phaeodactylum tricornutum* Grown in a Tubular Photobioreactor. J. Appl. Phycol..

[11] Guedes A.C., Meireles L.A., Amaro H.M., Malcata F.X. (2010). Changes in Lipid Class and Fatty Acid Composition of Cultures of *Pavlova lutheri*, in Response to Light Intensity. J. Am. Oil Chem. Soc..

[12] Hong S.J., Park Y.S., Han M.A., Kim Z.H., Cho B.K., Lee H., Choi H.K., Lee C.G. (2017). Enhanced Production of Fatty Acids in Three Strains of Microalgae using a Combination of Nitrogen Starvation and Chemical Inhibitors of Carbohydrate Synthesis. Biotechnol. Bioprocess Eng..

[13] Kamalanathan M., Pierangelini M., Shearman L.A., Gleadow R., Beardall J. (2016). Impacts of nitrogen and phosphorus starvation on the physiology of *Chlamydomonas reinhardtii*. J. Appl. Phycol..

[14] Lauritano C., De Luca D., Amoroso M., Benfatto S., Maestri S., Racioppi C., Esposito F., Ianora A. (2019). New molecular insights on the response of the green alga *Tetraselmis suecica* to nitrogen starvation. Sci. Rep..

[15] Venkata Mohan S., Rohit M.V., Chiranjeevi P., Chandra R., Navaneeth B. (2015). Heterotrophic microalgae cultivation to synergize biodiesel production with waste remediation: Progress and perspectives. Bioresour. Technol..

[16] Plaza M., Herrero M., Cifuentes A., Ibanez E. (2009). Innovative natural functional ingredients from microalgae. J. Agric. Food Chem..

[17] Mimouni V., Ulmann L., Pasquet V., Mathieu M., Picot L., Bougaran G., Cadoret J.P., Morant-Manceau A., Schoefs B. (2012). The potential of microalgae for the production of bioactive molecules of pharmaceutical interest. Curr. Pharm. Biotechnol..

[18] Lauritano C., Ferrante M.I., Rogato A. (2019). Marine natural products from microalgae: An -omics overview. Mar. Drugs.

[19] Mishra A., Medhi K., Malaviya P., Thakur I.S. (2019). Omics approaches for microalgal applications: Prospects and challenges. Bioresour. Technol..

[20] Heo S.J., Yoon W.J., Kim K.N., Oh C., Choi Y.U., Yoon K.T., Kang D.H., Qian Z.J., Choi I.W., Jung W.K. (2012). Anti-inflammatory effect of fucoxanthin derivatives isolated from *Sargassum siliquastrum* in lipopolysaccharide-stimulated RAW 264.7 macrophage. Food Chem. Toxicol..

[21] Kong Z.-L., Kao N.-J., Hu J.-Y., Wu C.-S. (2016). Fucoxanthin-Rich Brown Algae Extract Decreases Inflammation and Attenuates Colitis-associated Colon Cancer in Mice. J. Food Nutr. Res..

[22] Mosser D.M., Edwards J.P. (2008). Exploring the full spectrum of macrophage activation. Nat. Rev. Immunol..

[23] Yang S., Wan H., Wang R., Hao D. (2019). Sulfated polysaccharides from *Phaeodactylum tricornutum*: Isolation, structural characteristics, and inhibiting HepG2 growth activity in vitro. PeerJ.

[24] Shishido S.N., Varahan S., Yuan K., Li X.D., Fleming S.D. (2012). Humoral innate immune response and disease. Clin. Immunol..

[25] Medzhitov R. (2007). Recognition of microorganisms and activation of the immune response. Nature.

[26] Chan A.H., Schroder K. (2019). Inflammasome signaling and regulation of interleukin-1 family cytokines. J. Exp. Med..

[27] Cinelli M.A., Do H.T., Miley G.P., Silverman R.B. (2019). Inducible nitric oxide synthase: Regulation, structure, and inhibition. Med. Res. Rev..

[28] Pardoll D.M. (2012). The blockade of immune checkpoints in cancer immunotherapy. Nat. Rev. Cancer.

[29] Ben-Aharon O., Magnezi R., Leshno M., Goldstein D.A. (2018). Association of Immunotherapy With Durable Survival as Defined by Value Frameworks for Cancer Care. JAMA Oncol..

[30] Moynihan K.D., Opel C.F., Szeto G.L., Tzeng A., Zhu E.F., Engreitz J.M., Williams R.T., Rakhra K., Zhang M.H., Rothschilds A.M. (2016). Eradication of large established tumors in mice by combination immunotherapy that engages innate and adaptive immune responses. Nat. Med..

[31] Zhu E.F., Gai S.A., Opel C.F., Kwan B.H., Surana R., Mihm M.C., Kauke M.J., Moynihan K.D., Angelini A., Williams R.T. (2015). Synergistic innate and adaptive immune response to combination immunotherapy with anti-tumor antigen antibodies and extended serum half-life IL-2. Cancer Cell.

[32] Coffman R.L., Sher A., Seder R.A. (2010). Vaccine adjuvants: Putting innate immunity to work. Immunity.

[33] De Gregorio E., Rappuoli R. (2014). From empiricism to rational design: A personal perspective of the evolution of vaccine development. Nat. Rev. Immunol..

[34] Akinleye A., Rasool Z. (2019). Immune checkpoint inhibitors of PD-L1 as cancer therapeutics. J. Hematol. Oncol..

[35] Topalian S.L., Hodi F.S., Brahmer J.R., Gettinger S.N., Smith D.C., McDermott D.F., Powderly J.D., Carvajal R.D., Sosman J.A., Atkins M.B. (2012). Safety, activity, and immune correlates of anti-PD-1 antibody in cancer. N. Engl. J. Med..

[36] Morse M.A. (2005). Technology evaluation: Ipilimumab, Medarex/Bristol-Myers Squibb. Curr. Opin. Mol. Ther..

[37] Rebe C., Ghiringhelli F. (2019). STAT3, a Master Regulator of Anti-Tumor Immune Response. Cancers (Basel).

[38] Kwek S.S., Cha E., Fong L. (2012). Unmasking the immune recognition of prostate cancer with CTLA4 blockade. Nat. Rev. Cancer.

[39] Scott A.M., Allison J.P., Wolchok J.D. (2012). Monoclonal antibodies in cancer therapy. Cancer Immun..

[40] Shepard H.M., Phillips G.L., D Thanos C., Feldmann M. (2017). Developments in therapy with monoclonal antibodies and related proteins. Clin. Med..

[41] Lim W.A., June C.H. (2017). The Principles of Engineering Immune Cells to Treat Cancer. Cell.

[42] Ingebrigtsen R.A., Hansen E., Andersen J.H., Eilertsen H.C. (2016). Light and temperature effects on bioactivity in diatoms. J. Appl. Phycol..

[43] Lauritano C., Andersen J.H., Hansen E., Albrigtsen M., Escalera L., Esposito F., Helland K., Hanssen K.O., Romano G., Ianora A. (2016). Bioactivity Screening of Microalgae for Antioxidant, Anti-Inflammatory, Anticancer, Anti-Diabetes, and Antibacterial Activities. Front. Mar. Sci..

[44] Samarakoon K.W., Ko J.Y., Shah M.M.R., Lee J.H., Kang M.C., O-Nam K., Lee J.B., Jeon Y.J. (2013). In vitro studies of anti-inflammatory and anticancer activities of organic solvent extracts from cultured marine microalgae. Algae.

[45] Lavy A., Naveh Y., Coleman R., Mokady S., Werman M.J. (2003). Dietary Dunaliella bardawil, a beta-carotene-rich alga, protects against acetic acid-induced small bowel inflammation in rats. Inflamm. Bowel Dis..

[46] Newton K., Dixit V.M. (2012). Signaling in Innate Immunity and Inflammation. Cold Spring Harb. Perspect. Biol..

[47] Ebrahimi-Mameghani M., Sadeghi Z., Abbasalizad Farhangi M., Vaghef-Mehrabany E., Aliashrafi S. (2017). Glucose homeostasis, insulin resistance and inflammatory biomarkers in patients with non-alcoholic fatty liver disease: Beneficial effects of supplementation with microalgae *Chlorella vulgaris*: A double-blind placebo-controlled randomized clinical trial. Clin. Nutr..

[48] Caroprese M., Albenzio M., Ciliberti M.G., Francavilla M., Sevi A. (2012). A mixture of phytosterols from *Dunaliella tertiolecta* affects proliferation of peripheral blood mononuclear cells and cytokine production in sheep. Vet Immunol. Immunopathol..

[49] Cutignano A., Nuzzo G., Ianora A., Luongo E., Romano G., Gallo C., Sansone C., Aprea S., Mancini F., D’Oro U. (2015). Development and Application of a Novel SPE-Method for Bioassay-Guided Fractionation of Marine Extracts. Mar. Drugs.

[50] Guzman S., Gato A., Lamela M., Freire-Garabal M., Calleja J.M. (2003). Anti-inflammatory and immunomodulatory activities of polysaccharide from *Chlorella stigmatophora* and *Phaeodactylum tricornutum*. Phytother. Res..

[51] Barsanti L., Gualtieri P. (2019). Paramylon, a Potent Immunomodulator from WZSL Mutant of *Euglena gracilis*. Molecules.

[52] Russo R., Barsanti L., Evangelista V., Frassanito A.M., Longo V., Pucci L., Penno G., Gualtieri P. (2017). *Euglena gracilis* paramylon activates human lymphocytes by upregulating pro-inflammatory factors. Food Sci. Nutr..

[53] Bae S.Y., Yim J.H., Lee H.K., Pyo S. (2006). Activation of murine peritoneal macrophages by sulfated exopolysaccharide from marine microalga *Gyrodinium impudicum* (strain KG03): Involvement of the NF-kappa B and JNK pathway. Int. Immunopharmacol..

[54] Park G.T., Go R.E., Lee H.M., Lee G.A., Kim C.W., Seo J.W., Hong W.K., Choi K.C., Hwang K.A. (2017). Potential Anti-proliferative and Immunomodulatory Effects of Marine Microalgal Exopolysaccharide on Various Human Cancer Cells and Lymphocytes In Vitro. Mar. Biotechnol..

[55] Kwak J.H., Baek S.H., Woo Y., Han J.K., Kim B.G., Kim O.Y., Lee J.H. (2012). Beneficial immunostimulatory effect of short-term *Chlorella* supplementation: Enhancement of natural killer cell activity and early inflammatory response (randomized, double-blinded, placebo-controlled trial). Nutr. J..

[56] Chuang W.C., Ho Y.C., Liao J.W., Lu F.J. (2014). *Dunaliella salina* Exhibits an Antileukemic Immunity in a Mouse Model of WEHI-3 Leukemia Cells. J. Agric. Food Chem..

[57] Cerezuela R., Guardiola F.A., Meseguer J., Esteban M.A. (2012). Enrichment of gilthead seabream (*Sparus aurata* L.) diet with microalgae: Effects on the immune system. Fish Physiol. Biochem..

[58] De Jesus Raposo M.F., De Morais A.M., De Morais R.M. (2015). Marine polysaccharides from algae with potential biomedical applications. Mar. Drugs.

[59] Yim J.H., Kim S.J., Ahn S.H., Lee H.K. (2007). Characterization of a novel bioflocculant, p-KG03, from a marine dinoflagellate, *Gyrodinium impudicum* KG03. Bioresour. Technol..

[60] Hirahashi T., Matsumoto M., Hazeki K., Saeki Y., Ui M., Seya T. (2002). Activation of the human innate immune system by *Spirulina*: Augmentation of interferon production and NK cytotoxicity by oral administration of hot water extract of *Spirulina platensis*. Int. Immunopharmacol..

[61] Wu Q., Liu L., Miron A., Klimova B., Wan D., Kuca K. (2016). The antioxidant, immunomodulatory, and anti-inflammatory activities of *Spirulina*: An overview. Arch. Toxicol..

[62] Raposo M.F., De Morais R.M., Bernardo de Morais A.M. (2013). Bioactivity and applications of sulphated polysaccharides from marine microalgae. Mar. Drugs.

[63] Liu Y., Ma N., Sun X., Duan M., Luo T., Jiang P., Jiang G., Song S., Ai C. (2019). Effect of intake pattern of sulfated polysaccharides on its biological activity in high fat diet-fed mice. Int. J. Biol. Macromol..

[64] Muhamad I.I., Zulkifli N., Selvakumaran S.A., Lazim N.A.M. (2019). Bioactive Algal-Derived Polysaccharides: Multi-Functionalization, Therapeutic Potential and Biomedical Applications. Curr. Pharm. Des..

[65] Sun C., Liu M., Sun P., Yang M., Yates E.A., Guo Z., Fernig D.G. (2019). Sulfated polysaccharides interact with fibroblast growth factors and protect from denaturation. FEBS Open Bio.

[66] Vishwakarma J., Vavilala S.L. (2019). Evaluating the antibacterial and antibiofilm potential of sulfated polysaccharides extracted from green algae *Chlamydomonas reinhardtii*. J. Appl. Microbiol..

[67] Abdala Diaz R.T., Casas Arrojo V., Arrojo Agudo M.A., Cardenas C., Dobretsov S., Figueroa F.L. (2019). Immunomodulatory and Antioxidant Activities of Sulfated Polysaccharides from *Laminaria ochroleuca*, *Porphyra umbilicalis*, and *Gelidium corneum*. Mar. Biotechnol..

[68] Bahramzadeh S., Tabarsa M., You S., Li C., Bita S. (2019). Purification, structural analysis and mechanism of murine macrophage cell activation by sulfated polysaccharides from *Cystoseira indica*. Carbohydr. Polym..

[69] Cui J.F., Ye H., Zhu Y.J., Li Y.P., Wang J.F., Wang P. (2019). Characterization and Hypoglycemic Activity of a Rhamnan-Type Sulfated Polysaccharide Derivative. Mar. Drugs.

[70] Cao S., He X., Qin L., He M., Yang Y., Liu Z., Mao W. (2019). Anticoagulant and Antithrombotic Properties in Vitro and in Vivo of a Novel Sulfated Polysaccharide from Marine Green Alga *Monostroma nitidum*. Mar. Drugs.

[71] Da Silva F.R.P., Moara E.S.C.P., de Carvalho Franca L.F., Alves E.H.P., Dos Santos Carvalho J., Di Lenardo D., Brito T.V., Medeiros J.R., de Oliveira J.S., Freitas A.L.P. (2019). Sulfated polysaccharides from the marine algae *Gracilaria caudata* prevent tissue damage caused by ligature-induced periodontitis. Int. J. Biol. Macromol..

[72] Frentzen M. (2004). Phosphatidylglycerol and sulfoquinovosyldiacylglycerol: Anionic membrane lipids and phosphate regulation. Curr. Opin. Plant Biol..

[73] Kobayashi K. (2016). Role of membrane glycerolipids in photosynthesis, thylakoid biogenesis and chloroplast development. J. Plant Res..

[74] Roy A.B., Hewlins M.J., Ellis A.J., Harwood J.L., White G.F. (2003). Glycolytic breakdown of sulfoquinovose in bacteria: A missing link in the sulfur cycle. Appl. Environ. Microbiol..

[75] Speciale G., Jin Y., Davies G.J., Williams S.J., Goddard-Borger E.D. (2016). YihQ is a sulfoquinovosidase that cleaves sulfoquinovosyl diacylglyceride sulfolipids. Nat. Chem. Biol..

[76] Hielscher-Michael S., Griehl C., Buchholz M., Demuth H.U., Arnold N., Wessjohann L.A. (2016). Natural Products from Microalgae with Potential against Alzheimer’s Disease: Sulfolipids Are Potent Glutaminyl Cyclase Inhibitors. Mar. Drugs.

[77] Manzo E., Gallo C., Fioretto L., Nuzzo G., Barra G., Pagano D., Krauss I.R., Paduano L., Ziaco M., DellaGreca M. (2019). Diasteroselective Colloidal Self-Assembly Affects the Immunological Response of the Molecular Adjuvant Sulfavant. ACS Omega.

[78] Khozin-Goldberg I., Leu S., Boussiba S. (2016). Microalgae as a source for VLC-PUFA production. Subcell Biochem..

[79] Colombo S.M., Wacker A., Parrish C.C., Kainz M.J., Arts M.T. (2017). A fundamental dichotomy in long-chain polyunsaturated fatty acid abundance between and within marine and terrestrial ecosystems. Environ. Rev..

[80] Jacob-Lopes E., Maroneze M.M., Depra M.C., Sartori R.B., Dias R.R., Zepka L.Q. (2019). Bioactive food compounds from microalgae: An innovative framework on industrial biorefineries. Curr. Opin. Food Sci..

[81] Cui Y., Thomas-Hall S.R., Schenk P.M. (2019). *Phaeodactylum tricornutum* microalgae as a rich source of omega-3 oil: Progress in lipid induction techniques towards industry adoption. Food Chem..

[82] Adkins Y., Kelley D.S. (2010). Mechanisms underlying the cardioprotective effects of omega-3 polyunsaturated fatty acids. J. Nutr. Biochem..

[83] Le P.N.T., Desbois A.P. (2017). Antibacterial Effect of Eicosapentaenoic Acid against Bacillus cereus and *Staphylococcus aureus*: Killing Kinetics, Selection for Resistance, and Potential Cellular Target. Mar. Drugs.

[84] Albracht-Schulte K., Gonzalez S., Jackson A., Wilson S., Ramalingam L., Kalupahana N.S., Moustaid-Moussa N. (2019). Eicosapentaenoic Acid Improves Hepatic Metabolism and Reduces Inflammation Independent of Obesity in High-Fat-Fed Mice and in HepG2 Cells. Nutrients.

[85] Rupasinghe H.P., Erkan N., Yasmin A. (2010). Antioxidant protection of eicosapentaenoic acid and fish oil oxidation by polyphenolic-enriched apple skin extract. J. Agric. Food Chem..

[86] Garcia de Acilu M., Leal S., Caralt B., Roca O., Sabater J., Masclans J.R. (2015). The Role of Omega-3 Polyunsaturated Fatty Acids in the Treatment of Patients with Acute Respiratory Distress Syndrome: A Clinical Review. BioMed Res. Int..

[87] Miyata J., Arita M. (2015). Role of omega-3 fatty acids and their metabolites in asthma and allergic diseases. Allergol. Int..

[88] Nappo M., Berkov S., Massucco C., Di Maria V., Bastida J., Codina C., Avila C., Messina P., Zupo V., Zupo S. (2012). Apoptotic activity of the marine diatom *Cocconeis scutellum* and eicosapentaenoic acid in BT20 cells. Pharm. Biol..

[89] Yamada H., Umemoto T., Kakei M., Momomura S.I., Kawakami M., Ishikawa S.E., Hara K. (2017). Eicosapentaenoic acid shows anti-inflammatory effect via GPR120 in 3T3-L1 adipocytes and attenuates adipose tissue inflammation in diet-induced obese mice. Nutr. Metab..

[90] Onodera T., Fukuhara A., Shin J., Hayakawa T., Otsuki M., Shimomura I. (2017). Eicosapentaenoic acid and 5-HEPE enhance macrophage-mediated Treg induction in mice. Sci. Rep..

[91] Zapata-Gonzalez F., Rueda F., Petriz J., Domingo P., Villarroya F., Diaz-Delfin J., de Madariaga M.A., Domingo J.C. (2008). Human dendritic cell activities are modulated by the omega-3 fatty acid, docosahexaenoic acid, mainly through PPAR(gamma):RXR heterodimers: Comparison with other polyunsaturated fatty acids. J. Leukoc. Biol..

[92] Monk J.M., Liddle D.M., Cohen D.J., Tsang D.H., Hillyer L.M., Abdelmagid S.A., Nakamura M.T., Power K.A., Ma D.W., Robinson L.E. (2016). The delta 6 desaturase knock out mouse reveals that immunomodulatory effects of essential n-6 and n-3 polyunsaturated fatty acids are both independent of and dependent upon conversion. J. Nutr. Biochem..

[93] Han L., Song S., Niu Y., Meng M., Wang C. (2017). Eicosapentaenoic Acid (EPA) Induced Macrophages Activation through GPR120-Mediated Raf-ERK1/2-IKKbeta-NF-kappaB p65 Signaling Pathways. Nutrients.

[94] Bigogno C., Khozin-Goldberg I., Boussiba S., Vonshak A., Cohen Z. (2002). Lipid and fatty acid composition of the green oleaginous alga *Parietochloris incisa*, the richest plant source of arachidonic acid. Phytochemistry.

[95] Dunstan G.A., Volkman J.K., Barrett S.M., Leroi J.M., Jeffrey S.W. (1994). Essential Polyunsaturated Fatty-Acids from 14 Species of Diatom (Bacillariophyceae). Phytochemistry.

[96] Martins T.G., Odebrecht C., Jensen L.V., D’Oca M.G.M., Wasielesky W. (2016). The contribution of diatoms to bioflocs lipid content and the performance of juvenile *Litopenaeus vannamei* (Boone, 1931) in a BFT culture system. Aquac. Res..

[97] Nguyen H.M., Cuine S., Beyly-Adriano A., Legeret B., Billon E., Auroy P., Beisson F., Peltier G., Li-Beisson Y. (2013). The green microalga *Chlamydomonas reinhardtii* has a single omega-3 fatty acid desaturase that localizes to the chloroplast and impacts both plastidic and extraplastidic membrane lipids. Plant Physiol..

[98] Haigh W.G., Yoder T.F., Ericson L., Pratum T., Winget R.R. (1996). The characterisation and cyclic production of a highly unsaturated homoserine lipid in *Chlorella minutissima*. Biochim. Biophys. Acta Lipids Lipid Metab..

[99] Nappo M., Berkov S., Codina C., Avila C., Messina P., Zupo V., Bastida J. (2009). Metabolite profiling of the benthic diatom *Cocconeis scutellum* by GC-MS. J. Appl. Phycol..

[100] Renaud S.M., Thinh L.V., Lambrinidis G., Parry D.L. (2002). Effect of temperature on growth, chemical composition and fatty acid composition of tropical Australian microalgae grown in batch cultures. Aquaculture.

[101] Gouveia L., Coutinho C., Mendonca E., Batista A.P., Sousa I., Bandarra N.M., Raymundo A. (2008). Functional biscuits with PUFA-omega 3 from Isochrysis galbana. J. Sci. Food Agric..

[102] Adolf J.E., Place A.R., Stoecker D.K., Harding L.W. (2007). Modulation of polyunsaturated fatty acids in mixotrophic *Karlodinium veneficum* (Dinophyceae) and its prey, *Storeatula major* (Cryptophyceae). J. Phycol..

[103] Molino A., Martino M., Larocca V., Di Sanzo G., Spagnoletta A., Marino T., Karatza D., Iovine A., Mehariya S., Musmarra D. (2019). Eicosapentaenoic Acid Extraction from *Nannochloropsis gaditana* using Carbon Dioxide at Supercritical Conditions. Mar. Drugs.

[104] Ma X.N., Chen T.P., Yang B., Liu J., Chen F. (2016). Lipid Production from *Nannochloropsis*. Mar. Drugs.

[105] Wen Z.Y., Chen F. (2000). Production potential of eicosapentaenoic acid by the diatom *Nitzschia laevis*. Biotechnol. Lett..

[106] Zhang D.M., Wen S.M., Wu X., Cong W. (2018). Effect of culture condition on the growth, biochemical composition and EPA production of alkaliphilic *Nitzschia plea* isolated in the Southeast of China. Bioprocess Biosyst. Eng..

[107] Carvalho A.P., Pontes I., Gaspar H., Malcata F.X. (2006). Metabolic relationships between macro- and micronutrients, and the eicosapentaenoic acid and docosahexaenoic acid contents of *Pavlova lutheri*. Enzym. Microb. Technol..

[108] Mayer C., Come M., Ulmann L., Zittelli G.C., Faraloni C., Nazih H., Ouguerram K., Chenais B., Mimouni V. (2019). Preventive Effects of the Marine Microalga *Phaeodactylum tricornutum*, Used as a Food Supplement, on Risk Factors Associated with Metabolic Syndrome in Wistar Rats. Nutrients.

[109] Moheimani N.R., Borowitzka M.A. (2006). The long-term culture of the coccolithophore *Pleurochrysis carterae* (Haptophyta) in outdoor raceway ponds. J. Appl. Phycol..

[110] Li T., Xu J., Wu H., Jiang P., Chen Z., Xiang W. (2019). Growth and Biochemical Composition of *Porphyridium purpureum* SCS-02 under Different Nitrogen Concentrations. Mar. Drugs.

[111] Pezzolesi L., Pichierri S., Samori C., Totti C., Pistocchi R. (2017). PUFAs and PUAs production in three benthic diatoms from the northern Adriatic Sea. Phytochemistry.

[112] Blanchemain A., Grizeau D. (1999). Increased production of eicosapentaenoic acid by *Skeletonema costatum* cells after decantation at low temperature. Biotechnol. Tech..

[113] Vidoudez C., Pohnert G. (2012). Comparative metabolomics of the diatom *Skeletonema marinoi* in different growth phases. Metabolomics.

[114] Fabregas J., Otero A., Dominguez A., Patino M. (2001). Growth Rate of the microalga *Tetraselmis suecica* changes the biochemical composition of Artemia species. Mar. Biotechnol..

[115] Speranza L., Pesce M., Patruno A., Franceschelli S., De Lutiis M.A., Grilli A., Felaco M. (2012). Astaxanthin treatment reduced oxidative induced pro-inflammatory cytokines secretion in U937: SHP-1 as a novel biological target. Mar. Drugs.

[116] Galasso C., Corinaldesi C., Sansone C. (2017). Carotenoids from marine organisms: Biological functions and industrial applications. Antioxidants.

[117] Yoshida H., Yanai H., Ito K., Tomono Y., Koikeda T., Tsukahara H., Tada N. (2010). Administration of natural astaxanthin increases serum HDL-cholesterol and adiponectin in subjects with mild hyperlipidemia. Atherosclerosis.

[118] Hussein G., Nakamura M., Zhao Q., Iguchi T., Goto H., Sankawa U., Watanabe H. (2005). Antihypertensive and neuroprotective effects of astaxanthin in experimental animals. Biol. Pharm. Bull..

[119] Brendler T., Williamson E.M. (2019). Astaxanthin: How much is too much? A safety review. Phytother. Res..

[120] Novoveská L., Ross M.E., Stanley M.S., Pradelles R., Wasiolek V., Sassi J.F. (2019). Microalgal carotenoids: A review of production, current markets, regulations, and future direction. Mar. Drugs.

[121] Davinelli S., Nielsen M.E., Scapagnini G. (2018). Astaxanthin in skin health, repair, and disease: A comprehensive review. Nutrients.

[122] Grimmig B., Morganti J., Nash K., Bickford P.C. (2016). Immunomodulators as therapeutic agents in mitigating the progression of Parkinson’s disease. Brain Sci..

[123] Park J.S., Chyun J.H., Kim Y.K., Line L.L., Chew B.P. (2010). Astaxanthin decreased oxidative stress and inflammation and enhanced immune response in humans. Nutr. Metab..

[124] Lin K.H., Lin K.C., Lu W.J., Thomas P.A., Jayakumar T., Sheu J.R. (2015). Astaxanthin, a carotenoid, stimulates immune responses by enhancing IFN-γ and Il-2 secretion in primary cultured lymphocytes in vitro and ex vivo. Int. J. Mol. Sci..

[125] Davinelli S., Melvang H.M., Andersen L.P., Scapagnini G., Nielsen M.E. (2019). Astaxanthin from shrimp cephalothorax stimulates the immune response by enhancing IFN-γ, IL-10, and IL-2 secretion in splenocytes of *Helicobacter pylori*-infected mice. Mar. Drugs.

[126] Barzkar N., Tamadoni Jahromi S., Poorsaheli H.B., Vianello F. (2019). Metabolites from Marine Microorganisms, Micro, and Macroalgae: Immense Scope for Pharmacology. Mar. Drugs.

[127] Yanai H., Masui Y., Katsuyama H., Adachi H., Kawaguchi A., Hakoshima M., Waragai Y., Harigae T., Sako A. (2018). An Improvement of Cardiovascular Risk Factors by Omega-3 Polyunsaturated Fatty Acids. J. Clin. Med. Res..

[128] Neumann U., Derwenskus F., Gille A., Louis S., Schmid-Staiger U., Briviba K., Bischoff S.C. (2018). Bioavailability and safety of nutrients from the microalgae *Chlorella vulgaris*, *Nannochloropsis oceanica* and *Phaeodactylum tricornutum* in C57BL/6 mice. Nutrients.

[129] Vinayak V., Manoylov K.M., Gateau H., Blanckaert V., Hérault J., Pencréac’H G., Marchand J., Gordon R., Schoefs B. (2015). Diatom milking? A review and new approaches. Mar. Drugs.

[130] Vaz B.D.S., Moreira J.B., de Morais M.G., Costa J.A.V. (2016). Microalgae as a new source of bioactive compounds in food supplements. Curr. Opin. Food Sci..

[131] Ariede M.B., Candido T.M., Jacome A.L.M., Velasco M.V.R., de Carvalho J.C.M., Baby A.R. (2017). Cosmetic attributes of algae—A review. Algal Res..

[132] Bule M.H., Ahmed I., Maqbool F., Bilal M., Iqbal H.M.N. (2018). Microalgae as a source of high-value bioactive compounds. Front. Biosci. Sch..

[133] Wang H.M.D., Chen C.C., Huynh P., Chang J.S. (2015). Exploring the potential of using algae in cosmetics. Bioresour. Technol..

[134] Mourelle M.L., Gómez C.P., Legido J.L. (2017). The potential use of marine microalgae and cyanobacteria in cosmetics and thalassotherapy. Cosmetics.

[135] Koyande A.K., Chew K.W., Rambabu K., Tao Y., Chu D.-T., Show P.-L. (2019). Microalgae: A potential alternative to health supplementation for humans. Food Sci. Hum. Wellness.

[136] Schadendorf D., Ascierto P.A., Haanen J., Espinosa E., Demidov L., Garbe C., Guida M., Lorigan P., Chiarion-Sileni V., Gogas H. (2019). Safety and efficacy of nivolumab in challenging subgroups with advanced melanoma who progressed on or after ipilimumab treatment: A single-arm, open-label, phase II study (CheckMate 172). Eur. J. Cancer.

[137] Wang J., Shen T., Wang Q., Zhang T., Li L., Wang Y., Fang Y. (2019). The long-term efficacy of cytokine-induced killer cellular therapy for hepatocellular carcinoma: A meta-analysis. Immunotherapy.

[138] Maus M.V., Grupp S.A., Porter D.L., June C.H. (2014). Antibody-modified T cells: CARs take the front seat for hematologic malignancies. Blood.

[139] Apostolopoulos V., Thalhammer T., Tzakos A.G., Stojanovska L. (2013). Targeting antigens to dendritic cell receptors for vaccine development. J. Drug Deliv..

[140] Palucka K., Banchereau J. (2012). Cancer immunotherapy via dendritic cells. Nat. Rev. Cancer.

[141] Mellman I. (2013). Dendritic cells: Master regulators of the immune response. Cancer Immunol. Res..

[142] Rossi M., Young J.W. (2005). Human dendritic cells: Potent antigen-presenting cells at the crossroads of innate and adaptive immunity. J. Immunol..

[143] Hirano N., Butler M.O., Xia Z., Ansen S., von Bergwelt-Baildon M.S., Neuberg D., Freeman G.J., Nadler L.M. (2006). Engagement of CD83 ligand induces prolonged expansion of CD8(+) T cells and preferential enrichment for antigen specificity. Blood.

[144] Snider J.T., Brauer M., Kee R., Batt K., Karaca-Mandic P., Zhang J., Goldman D.P. (2019). The Potential Impact of CAR T-Cell Treatment Delays on Society. Am. J. Manag. Care.

[145] Lauritano C., Ianora A. (2018). Grand Challenges in Marine Biotechnology: Overview of Recent EU-Funded Projects. Grand Challenges in Marine Biotechnology.

[146] Rubin B.A. (1980). A note on the development of the bifurcated needle for smallpox vaccination. WHO Chronic.

[147] Montaser R., Luesch H. (2011). Marine natural products: A new wave of drugs?. Future Med. Chem..

[148] Cameron F., Whiteside G., Perry C. (2011). Ipilimumab: First global approval. Drugs.

[149] Seimetz D., Heller K., Richter J. (2019). Approval of First CAR-Ts: Have we Solved all Hurdles for ATMPs?. Cell Med..

[150] Alves C., Silva J., Pinteus S., Gaspar H., Alpoim M.C., Botana L.M., Pedrosa R. (2018). From Marine Origin to Therapeutics: The Antitumor Potential of Marine Algae-Derived Compounds. Front. Pharmacol..

[151] De Coêlho D.F., Tundisi L.L., Cerqueira K.S., da Silva Rodrigues J.R., Mazzola P.G., Tambourgi E.B., de Souza R.R. (2019). Microalgae: Cultivation Aspects and Bioactive Compounds. Braz. Arch. Biol. Technol..

[152] Serif M., Dubois G., Finoux A.L., Teste M.A., Jallet D., Daboussi F. (2018). One-step generation of multiple gene knock-outs in the diatom *Phaeodactylum tricornutum* by DNA-free genome editing. Nat. Commun..

[153] Nymark M., Sharma A.K., Sparstad T., Bones A.M., Winge P. (2016). A CRISPR/Cas9 system adapted for gene editing in marine algae. Sci. Rep..

[154] Park S.J., Nakagawa T., Kitamura H., Atsumi T., Kamon H., Sawa S., Kamimura D., Ueda N., Iwakura Y., Ishihara K. (2004). IL-6 regulates in vivo dendritic cell differentiation through STAT3 activation. J. Immunol..

